# *Neisseria meningitidis* W135, Turkey

**DOI:** 10.3201/eid1005.030572

**Published:** 2004-05

**Authors:** Levent Doganci, Mehmet Baysallar, Mehmet Ali Saracli, Gulsen Hascelik, Alaaddin Pahsa

**Affiliations:** * Gulhane Military Medical Academy, Ankara, Turkey; † Hacettepe University Medical School, Ankara, Turkey

**Keywords:** N. meningitidis W135, Turkey

## Abstract

We describe the first case of *Neisseria meningitidis* W135 meningitis in Turkey. The strain was genotypically unrelated to the clone (W)ET-37, isolated from Hajj pilgrims in 2000.

## The Case

A previously healthy 20-year-old serviceman experienced chills, headache, and vomiting 2 days before being admitted to the hospital in March 2003. On physical examination, neck stiffness, Kernig sign, Brudzinski sign, and temperature of 40°C were noted. The patient’s cerebrospinal fluid (CSF) was turbid with increased protein and pressure; leukocyte count was 4,500/μL. CSF culture grew *Neisseria meningitidis* in 24 hours. The strain was serogrouped as W135 by specific antiserum (Difco, Sparks, MD) in Hacettepe Medical School, Turkey, and confirmed by the Centers for Disease Control and Prevention (CDC, Atlanta, GA). Blood culture results were negative, and the patient had no petechial rash. He was treated with high-dose cefotaxime (3 g every 6 hours for 14 days) and made a full recovery.

For this isolate, both disk-diffusion and E-test methods using cefotaxime, penicillin, tetracycline, and ciprofloxacin were performed according to the criteria defined by the British Society for Antimicrobial Chemotherapy (*1*,*2*). Both methods were performed on Iso-Sensitest agar (Oxoid, Basingstoke, UK), supplemented with 5% defibrinated horse blood and nicotine adenine dinucleotide (Sigma, Taufkrichen, Germany). The isolate was susceptible to all of the antimicrobial agents (Table). The serviceman did not attend the Hajj and had no history of travel or contact with returning pilgrims.

**Table:** Susceptibility testing results of the isolate by E-test and disk-diffusion method

Antimicrobial agent tested	MIC (μg/mL)	Zone diameter (mm)
Penicillin	0.050	30
Cefotaxime	0.016	30
Tetracycline	1.000	23
Ciprofloxacin	0.002	32

## Conclusions

To the best of our knowledge, *N. meningitidis* W135 meningococcal disease has never been reported in Turkey. One W135 isolate from an asymptomatic carrier was reported in a child in 2001 (*3*). Globally, W135 strains are often isolated after intensive vaccination campaigns against serogroup A and C meningococci have been implemented (*4*). This patient’s vaccination certificate confirmed that he had received a bivalent (A+C) meningococcal vaccine 2 months earlier, at the beginning of his military training period. Turkish military vaccination campaigns have used the A+C polysaccharide vaccine successfully for a decade.

Multilocus enzyme electrophoresis, pulsed-field gel electrophoresis (PFGE), multilocus sequence typing, multilocus DNA fragment typing, and sequencing the 16S rRNA gene are new genotypic approaches to characterize *N. meningitidis* strains (*5*). This isolate was genotyped by using PFGE and 16S sequencing at CDC; both methods showed that it was a different subtype than the one associated with the Hajj pilgrimage in 2000 and 2001 (Figure).

**Figure:**
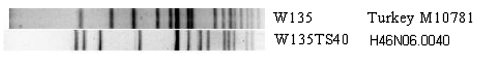
PFGE images of the case isolate (A) and the clone ET-37 (B).

In Turkey, most of the population is Muslim, and approximately 150,000 pilgrims travel annually to Saudi Arabia for the Hajj. During the pilgrimage in 2000 and 2001, an international outbreak was caused by a previously rare meningococcal serogroup W135 clone, (W)ET-37, possibly because conditions during the pilgrimage facilitate person-to-person transmission of meningococci (*6*,*7*). For the Hajj season of 2002 and 2003, all Turkish pilgrims received a quadrivalent meningococcal polysaccharide vaccine (Mencewax ACWY, SmithKline Beecham, Genval, Belgium). Although the quadrivalent meningococcal vaccine can protect persons against disease attributable to W135, it does not prevent them from becoming asymptomatic carriers, and therefore the vaccine may not prevent transmission to unvaccinated household contacts (*7*,*8*).

A recent study in the United States (*9*) showed that 0.8% of 727 returning pilgrims in 2001 were W135 carriers, although none had been on departure. To our knowledge, the rate of pilgrims returning to Turkey as W135 carriers has not been studied. On the basis of W135 transmission rates and epidemiologic data, we estimated the risk of an unvaccinated contact who had acquired W135 developing invasive meningococcal disease to be 1 case per 70 infections (*7*). In Singapore, disease usually developed within 14 days of a person’s contact with Hajj pilgrims, and no cases occurred 2 months after the end of Hajj pilgrimages (*7*). In Mauritius, a small tropical island in the Indian Ocean, one case of meningococcal disease caused by W135 occurred in a girl 3 months after her father returned from the Hajj pilgrimage; however, the virus could not be cultured, and it was not shown to be related to the Hajj strains (*10*). The case we report here occurred approximately 50 days after most Turkish pilgrims returned, which suggests that it was unrelated to the Hajj.

Although our patient had no history of travel or contact with a returning pilgrim, we investigated possible associations with the Hajj. However, PFGE results indicated that our patient’s strain was not closely related to the (W)ET-37 clone associated with the Hajj and may be unique to Turkey. Similarly, Jolley et al. from the Czech Republic have also reported sequence types of W135 other than (W)ET-37 (*11*). Additional investigation will be required to produce a database of well-documented Turkish cases. After the outbreaks in 2000 and 2001, many European countries reported additional cases of W135 meningitis in persons with no history of pilgrimage or contact with a returning pilgrim.

Since quadrivalent meningococcal vaccine does not prevent asymptomatic infection and therefore may not prevent returning pilgrims from transmitting W135 to unvaccinated household contacts, prophylactically administering antimicrobial agents should be considered to reduce the risk for transmission. Any decision to administer chemoprophylaxis to all returning pilgrims should depend on the rate of transmission of W135 infection from asymptomatic carriers to contacts after future pilgrimages. This case also showed the continuing need for administering quadrivalent meningococcal vaccine in the community. W135 meningococcal disease appears to be an emerging problem that should be investigated epidemiologically. This case confirmed the need to further study meningococcal carriers in order to build a national database and help make decisions on prophylaxis.
